# Research Advances in Pharmacology, Safety, and Clinical Applications of Yunnan Baiyao, a Traditional Chinese Medicine Formula

**DOI:** 10.3389/fphar.2021.773185

**Published:** 2021-11-24

**Authors:** Qi Yao, Bo-tao Chang, Rong Chen, Yi-jing Wei, Qiu-ju Gong, Dan Yu, Yang Zhang, Xu Han, Hong-bo Yang, Song-jiang Tang, Ying Gao

**Affiliations:** ^1^ Department of Anesthesiology, The First Affiliated Hospital of Guizhou University of TCM, Guiyang, China; ^2^ Department of Graduate, Guizhou University of TCM, Guiyang, China; ^3^ GLP Center, Yunnan Institute of Materia Medica, Kunming, China; ^4^ Department of Scientific Research, The First Affiliated Hospital of Guizhou University of TCM, Guiyang, China

**Keywords:** Yunnan Baiyao, pharmacology, safety, hemostasis, clinical applications

## Abstract

**Ethnopharmacology relevance:** Yunnan Baiyao (YNBY), a traditional Chinese medicine formulae, has some significant properties including activating blood circulation to dissipate blood stasis (Huo-Xue-Hua-Yu), eliminating swelling and alleviating pain (Xiao-Zhong-Zhi-Tong), and eliminating necrotic tissues and promoting granulation (Qu-Fu-Sheng-Ji).

**Aim of this study:** This paper intends to provide a comprehensive and critical analysis of studies on YNBY, proposing new possible therapeutic directions of this formula.

**Materials and methods:** Relevant data on YNBY were retrieved from available databases and a hand-search by searching the keywords such as “Yunnan Baiyao,” “pharmacology,” “toxicity,” and “clinical applications.”

**Results:** Traditionally, YNBY has been used to cure hemorrhage, bruises, swelling, and pain caused by injuries in the Chinese folk. Modern pharmacological studies show that YNBY possesses pharmacological activities including hemostasis, invigorating the circulation of blood, wound healing, anti-inflammation, analgesia, antibiosis, infection prevention, and other effects. Toxicological studies demonstrate that YNBY has a certain toxicology, which is mainly caused by Aconitum alkaloids from Cao-wu (CW, *Aconiti Kusnezoffii* Radix). The developmental non-toxic reaction dose (NOAEL) of YNBY for embryos and fetuses is 0.5 g/kg in rats. In addition, the NOAEL for fertility and early embryo development toxicity is 4.0 g/kg in rats. Clinical trials have confirmed the safety of YNBY in a large number of patients, and adverse drug reactions (ADRs) such as abdominal pain, diarrhea, allergy, and others in very few people. YNBY is routinely used in clinic to cure bleeding, pain, swelling, upper digestive tract ulcer, postoperative wound, arthritis, mouth ulcers, ulcerative colitis, etc.

**Conclusions:** Hemostasis is a conspicuous effect of YNBY. Except for this effect, analgesia and anti-infection may be new research directions of this formula. In addition, the *in vitro* and *in vivo* pharmacology and mechanisms of action of YNBY are encouraged as well as the pharmacokinetics of this formulae. Furthermore, the material basis of the pharmacological effects of YNBY also needs clear identification.

## Introduction

**FIGURE 1:**
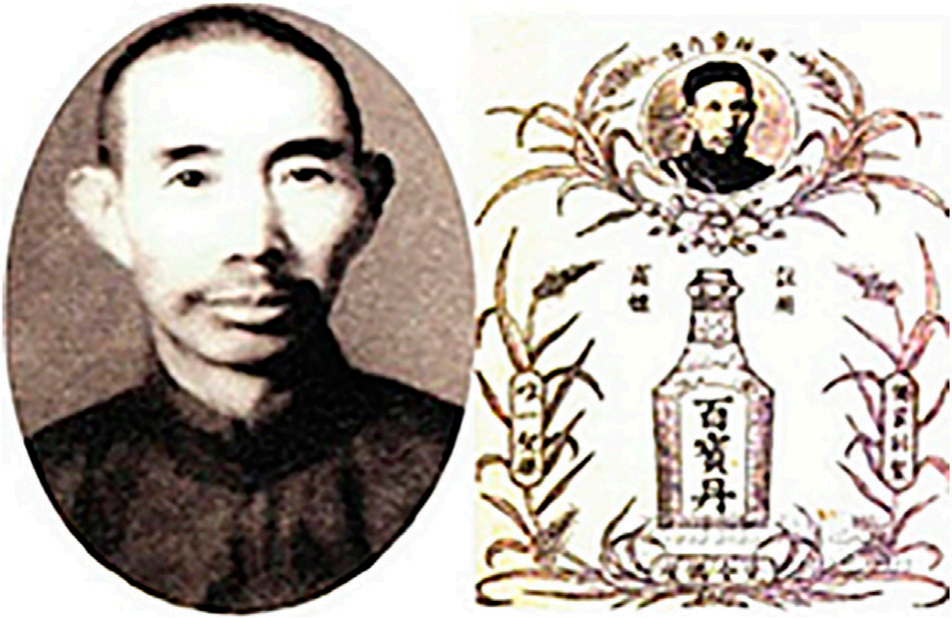
Huan-zhang Qu and the brand of Yunnan Baiyao during late Qing Dynasty (1840–1912) and the Republic of China (1912–1949).

Yunnan Baiyao (YNBY), a traditional Chinese medicine formulae originally called Qu-huan-zhang-wang-ying-bai-bao pill, was invented by a Chinese medicine doctor named Huan-zhang Qu (1880–1938) in 1902 (Figure 1).

YNBY shows significant hemostasis effects in front of bleeding caused by various factors including trauma, surgery, bronchiectasis and tuberculosis, gastric ulcer perforation, and delivery.

After the July 7 incident in 1937, the Yunnan troop of the Republic of China went north to fight against the Japanese invaders. Doctor Qu donated 30,000 bottles of YNBY to the Chinese army. In the Battle of Tai-er-zhuang, YNBY became an indispensable medicine for the wounded soldiers (Gao, 2002). After the founding of the People’s Republic of China, in 1955 doctor Qu’s wife Lan-ying Miu donated the secret prescription for YNBY to the new China without any compensation (Gao, 2002).

**FIGURE 2:**
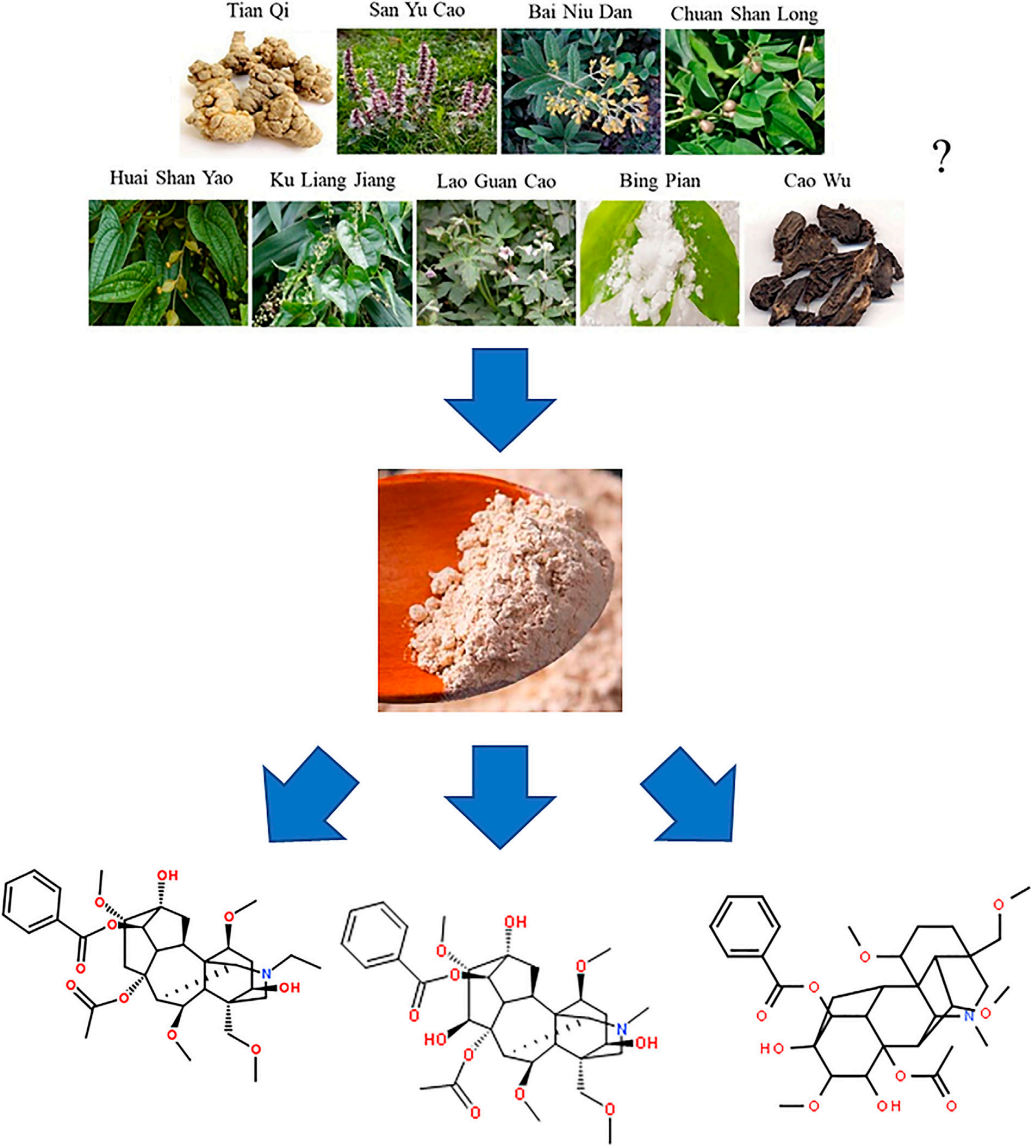
The possible prescription of Yunnan Baiyao.

Until now, the detailed prescription of this formula is still unknown to the public in China. In 2002, an American company named Herbmax submitted the related documents of the YNBY tincture as a dietary supplement to the US food and drug administration (FDA) for sale. Although this request was eventually rejected by the FDA, the documents clearly listed all the components in the YNBY tincture (Figure 2). However, the compatibility of these components was not shown in this list. Further, the poisonous herb Cao-wu was not mentioned although the existence of this herb has been widely accepted in the domestic Chinese medicine community. Therefore, the accuracy of the provided prescription of YNBY was doubtful.

In 2013, the Department of Health, Hong Kong Special Administrative Region, China, issued a press bulletin that the prescription of YNBY contained aconitum alkaloids undeclared. Therefore, the drug importer Feng-Hua Company in Hong Kong recalled five types of YNBY preparations. After that, the YNBY group issued an official statement and acknowledged the existence of CW in the prescription. Meanwhile, this statement noted that the toxicity of CW was eliminated or attenuated when the aconitine components were hydrolyzed into benzoyl aconitum by some special processing technologies (Ping, 2014).

In 2014, the YNBY group revised its drug instructions in accordance with the requirements of the National Medical Products Administration (NMPA) and officially declared that the formulae contained CW. Subsequently, the YNBY group reiterated the safety and efficacy of this formula when aconitine was hydrolyzed into hypaconitine and further hydrolyzed into benzoyl aconitine through processing technologies. Furthermore, some clinical trials also confirmed the clinical safety of YNBY in a large number of patients (Xu et al., 2018; Yang et al., 2018; Song et al., 2019; Xiao et al., 2019; Li et al., 2019).

CW is an important Chinese herbal medicine in YNBY. Traditionally, it has been used for dispelling wind and removing dampness, as well as eliminating swelling and alleviating pain. Routinely, CW is applied in clinic to cure joint pain, cold hernia, rheumatoid arthritis, and other diseases. Aconitine-type alkaloids including aconitine, neoaconitine, hypoaconitine, benzoyl aconitine, benzoyl neoaconitine, and benzoyl hypoaconitine were the main components in CW (Shao et al., 2016). They were both active and toxic ingredients because of a quite narrow range of safe doses. Li et al. (2018) reported 12 diterpene alkaloids in CW, including neoline, songorine, bewudine, hokbusine A, mesaconitine, mesaconine, talasamine, bewutine, songoramine, isotalatizidine, 8-methoxy-14-benzoyl-beiwutinine, and 8-OEt-14-benzoylmesaconine.

In addition, carbohydrates in CW mainly included glucose (Glc), galactose (Gal), and mannose (Man), and xylose (Xyl) (Zhang et al., 2016).

Ren et al. (2020b) confirmed that the YNBY treatment regulated five core toxicity biomarkers including prostaglandin E3, γ-glutamylleucine, 2-keto-6-acetamidocaproate, 3,4-dihydroxyphenylglycol O-sulfate, and 4-hydroxy-5-(3′-hydroxyphenyl)-valeric acid-3′-O-sulfate to normal condition using network pharmacology in combination with metabolomics. Moreover, the core metabolic pathway lysine degradation contributed to the detoxification of YNBY. Meanwhile, ACHE, SLC6A3, and SLC6A4 might be the targets of protective effects of other herbs in YNBY. In summary, these findings contributed greatly to the safety evaluation of YNBY.

**FIGURE 3:**
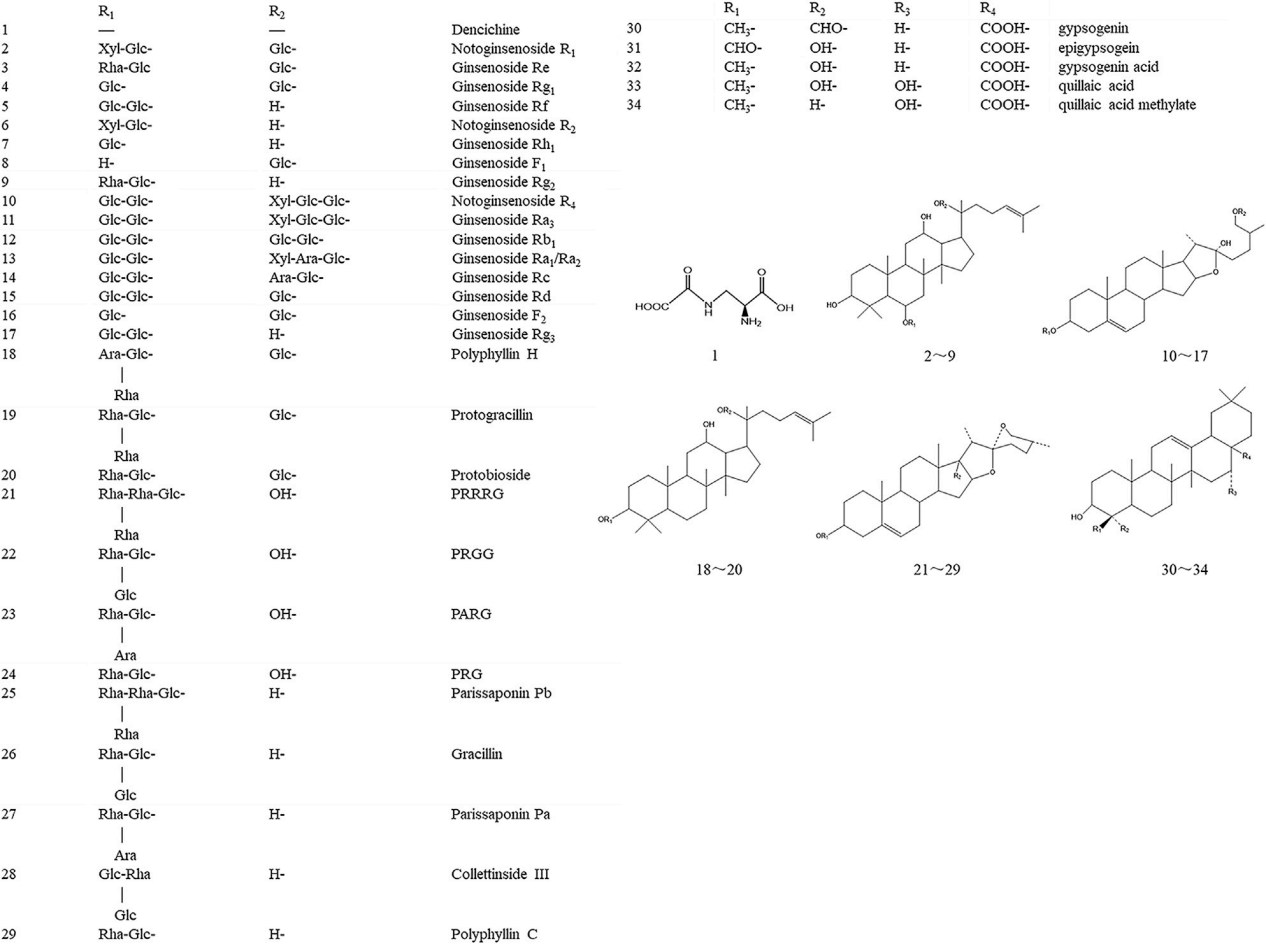
Chemical structures and names of the 34 compounds isolated and identified from Yunnan Baiyao. Glc, b-D-glucose; Rha, a-L-rhamnose; Xyl, b-D-xylose; Ara, a-L-arabinose. PRRRG: pennogenin-3-O-α-L-rhamnopyranosyl(1→4)-α-L-rhamnopyranosyl(1→4)-[α-L-rhamnopyranosyl (1→2)]-β-D-glucopyranoside; PRGG: pennogenin-3-O-α-L-rhamnopyranosyl(1→2)-[β-D-glucopyranosyl(1→3)]-β-D-glucopyranoside; PARG: pennogenin-3-O-α-L-rhamnopyranosyl(1→2)-[α-L-arabinopyranosyl(1→4)]-β-D-glucopyranoside; PRG: pennogenin-3-O-α-L-rhamnopyranosyl(1→2)-β-D-glucopyranoside.

Current chemical studies on YNBY are few. Dai et al. (2013) globally determined and identified 34 components from YNBY by liquid chromatography hybrid ion trap time-of-flight mass spectrometry (Figure 3). Furthermore, 13 compounds (2–4, 12, 16, 21–28) were selected to evaluate the quality of 27 YNBY samples (Liu et al., 2008).

Nowadays, YNBY exhibits procoagulant, wound healing, anti-inflammatory, analgesic, antibacterial, antitumor, and other effects. However, the mechanisms are still absent or unclearly clarified in most of the pharmacological studies. Due to national secrecy for prescription of traditional Chinese medicine (TCM) formulae, the active ingredients in YNBY as well as their structure–activity relationships (SARs) are still unknown. Although the YNBY toxicity has been reported in some experimental animals, it is necessary to investigate the toxicity of this formula in human beings systemically. In addition, YNBY has been used in clinic to cure bleeding, tissue injuries, or inflammation- or infection-related diseases in patients. Some adverse drug reactions (ADRs) occur in a small sample of cases. Therefore, it needs clarification of the correlation between the ADRs and YNBY, and adoption of some efficient measures to solve the ADRs.

This review manages to provide a critical and comprehensive analysis on the pharmacology, safety, and clinical applications of YNBY. In view of this, some evidence for the efficacy and safety of this TCM formula is provided to treat diseases in clinic. Moreover, this study will provide some new research directions for this formula in future.

## Pharmacological Effects

The pharmacological effects of YNBY have been investigated. The pharmacological effects are shown in Table 1.

**TABLE 1:** Pharmacological effects of YNBY.

Effects	Human/animal/cell	Dose and duration	Changes in the parameters measured	References
Hemostatic effects	Beagle dogs	1,000 mg every 12 h for five times	Control: 24-h MA↓, G↓ vs. baseline	Frederick et al. (2017)
			24-h LY30 ↑, LY60 ↑ vs. baseline	
			YNBY: 24-h LY30↑, LY60↑ vs. baseline	
	Dogs	250 mg twice daily for 1 week	YNBY: A30 ↑, A60 ↑, G↑, LY30↓, LY60↓ vs. control	Tansey et al. (2018)
	SD rats	340 and 670 mg/kg twice daily for 3 days	YNBY: PRP↑ (AA or ADP activation), CD61↑, CD62P↑, platelet activation percentage↑ (ADP activation) vs. control	Ye et al. (2004)
	Horses	15 mg/kg every 12 h for 3 days	No significant differences in 15 variables determined	Ness et al. (2017)
Invigorating the circulation of blood effects	Male SD rats	180 mg/kg for 15 days	YNBY: whole viscosity↓, PT↑, plasma fibrinogen↓, blood stasis↓, congestion↓, edema↓, inflammatory cell infiltration↓ vs. model	Yang et al. (2019a)
	Operative patients	0.5 g for 4 times daily for 3 days	YNBY: GPⅡb↑, GPⅢ140↑ vs. model	Zhang et al. (2004)
Wound healing effects	SD rats	4 mg once	YNBY: bone density↑, bFGF↑, BMP-2↑ vs. control	Liang, 2010
	HDPCs	1.25, 2.5, 5, 10, 20, 40 μg/ml for 3, 7, 14 days	YNBY: OC protein↑, Col-I mRNA↑, BSP mRNA↑ vs. control	Liu, (2014)
	Female C57BL/6 mice	100 μl 1% solution once	YNBY: wound healing↑, bioelectricity at the edge of wound↓, Na+↓, Ca2+↓, Cl-↓ vs. control	Sun, (2015)
Anti-inflammatory effects	SD rats	1 ml (50 mg/ml) for 7 days	YNBY: paw edema↓ (carrageenan- or AA-induced), cPLA2, FLAP, 5-LOX, and LTB4r mRNAs (AA-induced)↓, LTB4r, cPLA2 and 5-LOX proteins↓ (AA-induced), PGE2, cPLA2, COX-2 mRNAs and proteins↓ (AA-induced) vs. model	Ren et al. (2017)
	Osteoclasts	10, 50, and 200 μg/ml for 12, 24, 48 h	YNBY: IL-1β, IL-6, TNF-α proteins↓, COX-1, COX-2, A2, 5-LOX, sEH mRNAs↓, CYP2C8 and CYP2C9 mRNAs↑ vs. model	Ren et al. (2020c)
Antioxidant effects	—	0.2–1 mg/ml for 0.5 h	YNBY: DPPH↓, hydroxyl free radicals↓ vs. control	Li et al. (2016a)
Anti-arthritis effects	SD rats	1 ml (50 mg/ml) once a day for 4 weeks	YNBY: average voix pedis thickness↓, IL-1β↓, PGE2↓, inflammatory cell infiltration↓, synovium hyperplasia↓, narrowed joint space↓ vs. model	Luo et al., 2017
Antibacterial effects	HAPU patients	Appropriate amount for 20 days	YNBY: hla promoter activity↓, overall fluorescence intensity and fluorescence intensity of the largest biofilm↓, coagulase activity↓, enterotoxin and tsst-1↓, adhesion function↓ vs. untreated	Liu et al. (2019)
	*P. aeruginosa*	2.0, 2.5, 3.0 mg/ml for 2–24 h	YNBY: cell density, total bacterial and extracellular protein↓, *lasR*, *lasI*, *rhlR*, and *rhlI* mRNAs↓, LasA protease, LasB elastase, pyocyanin mRNAs↓, motility of *P. aeruginosa*↓ vs. control	Zhao et al. (2013)
Analgesic effects	Mice	Intraperitoneal injections of compounds (**1′-8′**) ranging 0.5–1 mg/kg once	YNBY: paw edema↓, number of writhes↓ vs. model	Li et al. (2016b)
Antitumor effects	Canine HSA cell lines	50, 100, 200, 400, 600 and 800 μg/ml for 24, 48, 72 h	YNBY: Caspase-3/7 activity↑, G1- and G2-phases↑, S-phase↓, VEGF↑ vs. control	Wirth et al. (2016)
Sleep-enhancing effects	ICR mice	0.3, 1.2, 4.8 g/kg for one time	YNBY: sleeping time↑ vs. vehicle control	Lu et al. (2014)

### Hemostatic Effects

Hemostasis is a most significant property of YNBY. Studies have indicated that YNBY significantly promotes platelet aggregation and shortens prothrombin time (PT), bleeding time, and clotting time (Frederick et al., 2017; Lee et al., 2017; Ness et al., 2017; Tansey et al., 2018; Patlogar et al., 2019) in the experimental animals.

In the study of Frederick et al. (2017), eight beagle dogs were administrated with placebo or 1,000 mg YNBY every 12 h for five times. The blood samples were collected 24 h prior to the administrations and 2 and 24 h post the last administrations followed by measurements of buccal mucosal bleeding times (BMBTs) immediately and whole-blood thromboelastography (TEG) analysis 60 ± 5 min after collecting the blood samples. It showed no adverse effects and significant differences in BMBTs or TEG parameters between the placebo group and the YNBY treatment group. Between baseline and 24 h post the last treatment, there were significant differences in the TEG parameters such as percentage clot lysis at 30 min (LY30) and at 60 min (LY60) within the YNBY-treated group, and TEG parameters including maximum amplitude (MA), shear elastic modulus strength (G), LY30, and LY60 within the control group. It suggested that YNBY had no significant effects on the coagulation-related parameters. Further, platelet function is to be determined directly in a larger number of samples.

Similarly, 18 healthy dogs were orally given 250 mg YNBY twice daily for 1 week. The TEG was analyzed prior to the treatment and post the last treatment. During this period, any side effects were observed and recorded (Tansey et al., 2018). The results revealed that the oral administration of YNBY significantly increased A30 and A60 as well as G. Meanwhile, LY30 and LY60 were significantly decreased at week 1 after the last treatment. Mild diarrhea was observed in only one dog after the oral administration of YNBY. These findings suggested that the oral YNBY increased the strength of the clot in the healthy dogs, and YNBY was well tolerant in most of the animals, which was consistent with the study of Frederick et al. (2017) in some degree. However, different disease statuses are warranted to further evaluate these preliminary findings in larger prospective studies.

The effects of YNBY on the expressions of platelet membrane glycoproteins were assayed in rats (Ye et al., 2004). Briefly, 30 rats were randomly separated into three groups (*n* = 10, half male and half female), including a control group and two YNBY-treated groups (340 and 670 mg/kg). The rats in the YNBY-treated groups were administrated with YNBY twice daily for 3 days. Correspondingly, the animals in the control group were orally given NS. One hour after the last administrations, all the rats were anaesthetized by intraperitoneal injections of 1% pentobarbital sodium (4 ml/kg). The external carotid artery on one side was separated, and 4.5 ml anticoagulant blood with 3.8% sodium citrate was taken using a disposable syringe. After that, platelet-rich plasma (PRP) and platelet-poor plasma (PPP) were separated routinely. Then, 300 μl PRP was put into a test cup and then the platelet aggregation rate at different time points and the maximum aggregation rate activated by arachidonic acid (AA, 4.3 mmol/l) or adenosine diphosphate (ADP, 10 μmol/l) were measured using a platelet function analyzer. The expressions of platelet membrane glycoproteins in the PRP-free blood samples were detected by fluorescence-activated cell sorting (FACS) under resting or ADP-activated status. The results showed that whether activated by AA or ADP, the aggregation rates of PRP at 120, 180, 240, and 300 min and the maximum aggregation rate within 6 min were significantly higher in the two YNBY-treated groups than those in the control groups (*p* < 0.05 for all analyses) (Table 2). Interestingly, the expressions of CD61 and CD62P and the platelet activation percentage were remarkably higher in these two YNBY-treated groups than those in the control group after being activated by ADP (*p* < 0.01 for all analyses) (Table 3).

**TABLE 2:** Platelet aggregation rates and maximum aggregation rate after treated with YNBY under AA- or ADP-activated status ( $\bar{\text{x}}$ ± s, *n* = 10).

Group	Aggregation rate (%)										Maximum aggregation rate (%)	
	60 min		120 min		180 min		240 min		300 min					
	AA	ADP	AA	ADP	AA	ADP	AA	ADP	AA	ADP	AA	ADP
Control	7.6 ± 6.4	12.6 ± 12.1	15.2 ± 14.8	24.6 ± 12.4	23.6 ± 19.3	34.8 ± 14.7	27.0 ± 20.6	41.7 ± 18.2	31.4 ± 19.7	54.6 ± 21.9	49.1 ± 9.9	57.2 ± 21.7
340 mg/kg	23.5 ± 12.9^a^	14.7 ± 6.3	39.5 ± 18.2^a^	32.6 ± 7.9	43.6 ± 14.5^b^	51.3 ± 10.1^a^	51.5 ± 8.8^a^	72.7 ± 12.5^a^	60.8 ± 8.8^a^	76.7 ± 10.3^a^	66.3 ± 10.2^a^	88.6 ± 11.4^a^
670 mg/kg	27.8 ± 20.1^b^	15.6 ± 9.8	44.3 ± 23.1^b^	36.8 ± 7.7^b^	51.6 ± 20.4^b^	57.3 ± 9.7^a^	54.5 ± 17.1^a^	77.3 ± 13.9^a^	60.8 ± 14.5^a^	88.0 ± 14.8^a^	66.4 ± 12.2^a^	92.2 ± 11.2^a^

a *p* < 0.05 vs. control.

b *p* < 0.05.

**TABLE 3:** Expressions of platelet membrane glycoproteins after being treated with YNBY under resting or ADP-activated status ( $\bar{\text{x}}$ ± s, *n* = 10).

Group	Resting			ADP-induced		
	CD61 (MFI)	CD62P(MFI)	Activation percentage (%)	CD61(MFI)	CD62P(MFI)	Activation percentage (%)
Control	33.6 ± 6.1	3.5 ± 0.8	10.3 ± 4.6	44.3 ± 6.0	5.1 ± 1.2	24.8 ± 7.0
340 mg/kg	35.2 ± 4.8	4.1 ± 0.5	13.6 ± 3.7	57.3 ± 10.6^b^	7.9 ± 1.7^b^	40.0 ± 7.9^b^
670 mg/kg	37.4 ± 6.1	4.2 ± 0.6	12.5 ± 3.9	61.0 ± 13.0^b^	8.2 ± 2.4^b^	39.6 ± 8.7^b^

a *p* < 0.05.

b *p* < 0.05 vs. control; MFI: median fluorescence intensity.

When the platelet is activated, CD61 transfers from the inside to the outside of the membrane through the platelet open duct system, which increases its expression on the plasma membrane (Goodall and Appleby, 2004). Meanwhile, its binding to CD41 also increased, and the configuration of the CD41/CD61 complex is changed, which enhances the binding to the fibrinogen receptor and promotes the platelet aggregation (Larsson et al., 2002). The platelet α granule membrane fuses with the plasma membrane, which exposes CD62P as a marker of late platelet activation on the surface (Valkonen et al., 2019).

This study showed no significant differences in the CD6 and CD62P expressions and the platelet activation percentage upon the resting status among the groups. However, under the ADP-activated status, the reaction of the platelet was more sensitive after the YNBY treatments and the activation and expressions of glycoproteins were significantly enhanced as compared with the control. More glycoproteins were expressed on the membrane surface and participated in the aggregation and adhesion of the platelets, which contributed to the hemostatic effects of this formula. These results partly provided some experimental basis for YNBY-reducing bleeding under some specific status such as postoperation. Although this study preliminarily reveals the hemostatic effects of YNBY, the procoagulant mechanism of this formula has not been clearly clarified yet.

In addition, a randomized blinded placebo-controlled crossover study on YNBY was performed in 12 healthy adult horses (Ness et al., 2017). The horses were orally administered with a paste containing YNBY (15 mg/kg) or a placebo paste every 12 h for three consecutive days. Zero, 24, and 72 h after the administrations, blood samples were collected for measurements. The results showed no significant differences in 15 variables including platelets, clotting time, vWF:Ag, vWF:Ag to vWF:RCo, ADPMA, PselTH, thrombin-stimulated phosphatidylserine externalization, PMPTH, PLleuk, aPTT, PT, fibrinogen, tPACLT, HCT, and α angle between the YNBY-treated group and the placebo group at any time point (*p* > 0.05 for all analyses). It suggests that a typical dose of YNBY in clinic has no significant effects on platelet or vWF function, as well as fibrin-clot formation or stability. Therefore, according to the previous studies (Ye et al., 2004; Frederick et al., 2017), higher doses of YNBY may be required or hemostatic effects are to be determined in the presence of vascular and/or tissue injury.

### Invigorating the Circulation of Blood Effects

On the one hand, YNBY exerts its hemostatic effects. On the other hand, this formula improves blood circulation (Yang et al., 2019a).

Thirty-three male SPF SD rats (9 weeks old) weighing 250 ± 20 g were randomly divided into a blank control group, a model group, and a YNBY group (*n* = 11). The rats in the model group were subcutaneously injected with adrenalin hydrochloride (0.3 mg/kg) daily for seven consecutive days. The rats in the YNBY-treated group as well as the model group were injected with adrenalin hydrochloride. After that, the animals in the YNBY-treated group were administrated with 180 mg/kg YNBY for 15 consecutive days. At the end of the experiment, the animals were anesthetized by intraperitoneal injections of 3% pelltobarbitalum natricum (1 ml/kg) and then punctured abdominal aortas and collected blood samples for the tests. Compared with the model group, YNBY significantly reduced the whole viscosity when the shear rates were 3, 100, and 180 s^−1^, respectively (*p* < 0.05; *p* < 0.01; *p* < 0.05). The YNBY treatment markedly prolonged the plasma prothrombin time (PT) and reduced the plasma fibrinogen level in the blood stasis symptom-complex (BSSC) rats compared to the model (*p* < 0.05; *p* < 0.05). Histopathological findings showed that the YNBY treatment efficiently improved the pathological conditions such as blood stasis, congestion, edema, and inflammatory cell infiltration in various organs including heart, livers, spleens, lungs, and kidneys of the rats with BSSC. Then, UPLC-Synapt G2-Si was used to collect the serum data and then input into the Progenesis QI software for processing. Subsequently, the obtained molecular and ionic information was matched with a self-built CW component database to identify those unknown compounds by analyzing their secondary information and fragmentation patterns. The serum chromatograms were assayed and compared, and then the compounds were matched with the preestablished CW database. Finally, seven migrating components of aconitum alkaloids in the blood were identified to be karakoline, denudatine, karacolidine, isotalatizidine, senbusine B, talatisamine, and chasmanine, which contributed to CW relieving adrenalin hydrochloride-induced BSSC. However, some limitations were obvious in the present study. If the modeling success rate is not 100%, it is necessary to select the animals with BSSC according to the related standards prior to the grouping. Additionally, it is far from enough to reflect the dose–efficacy relationship because only a single dose of YNBY was used in the *in vivo* experiment. Moreover, the positive control drug was absent in the experiment and a common platelet aggregation inhibitor such as aspirin may be an appropriate choice.

YNBY exhibits not only hemostatic effects (Zhi-xue in the TCM concept) but also invigorates the circulation of blood effects (Huo-xue in the TCM concept). Zhang et al. (2014) investigated the effect of YNBY on the ultrastructure and perioperation surface glycoprotein of platelets. Briefly, 120 operative patients were randomly separated into three groups (*n* = 40), including a control group, a tumor surgery group, and a non-tumor surgery group. The double-blind method was used in the study. The patients in the two experiment groups orally took 0.5 g YNBY for four times daily for three consecutive days. Correspondingly, the patients in the control group took empty capsules. FACS was used to determine the expressions of GPⅡb, GPⅢa, and GMP140 before taking this medicine, before and after the operation. Meanwhile, ultrastructures of D-dimer and platelet were observed. The results indicated that YNBY markedly increased the expressions of platelet GPⅡb and GPⅢ140 (*p* < 0.01; *p* < 0.01), but it had no significant effect on the D-dimer-positive rate (*p* > 0.05). Electron microscopy showed that YNBY remarkably elevated activated platelets with increased platelet surface protrusions, platelet tubules’ opening, and releasing. In summary, taking the specific dose of YNBY could increase the platelet activity, platelet aggregation, and its ability to bind to fibrinogen receptors, and it brought no risk of thrombosis during the perioperative period.

### Wound Healing Effects

The wound healing effects of YNBY partly support the eliminating necrotic tissues and promoting granulation. Some experimental studies have confirmed its wound healing effects (Liang, 2010; Liu, 2014; Sun, 2015; Yan, 2015), which was closely related to the upregulations of some growth-related factors such as bFGF, BMP-2, and VEGF (Liang, 2010; Yang et al., 2011).

Liang (2010) evaluated the healing effects of YNBY in rats after cleft palate surgery. Briefly, 90 SPF SD rats (3–4 months) weighing 300 ± 10 g (genders not referred) were allocated into three groups: a control group, a triiodomethane group, and a YNBY group. The cleft palate model in rats was prepared by removing a palatine bone (depth: 2.0 mm, length: 7.0 mm, width: 2.5 mm) using an electric bone drill. Four milligrams of YNBY or 4 mg triiodomethane was applied in the prepared bone defects, respectively. No drug was applied in the control group. After that, the wounds were sutured with 000 sutures. During the operation, it was YNBY not triiodomethane that showed significant hemostatic effects. A radiographic examination of the damage in the palate bone showed callus formation, defect area connection, and continuity of palatine bone in the YNBY-treated group 3 weeks after the operation. The bone defect was completely filled with callus in the YNBY group, and the bone density was close to the surrounding bone tissue. In addition, the bone density in the YNBY-treated group was respectively higher than that in the triiodomethane group and the control group (3rd week: 0.397 ± 0.027 vs. 0.354 ± 0.016 vs. 0.357 ± 0.025; 4th week: 0.441 ± 0.019 vs. 0.398 ± 0.021 vs. 0.396 ± 0.018. *p* < 0.05 for all the analyses). Four weeks after the operation, the pathology revealed that in the YNBY-treated group the defect areas were filled with bony callus mainly lamellar bone. The bone lacunas were observed, and they were not distinct from the surrounding normal bone tissues clearly. Modified Gomori trichrome staining showed that the bone tissues in the YNBY group were continuous and the periostea were intact and similar to the normal tissues 4 weeks after the operation. Immunohistochemistry analysis revealed that YNBY and triiodomethane significantly increased the positive bFGF expression represented by optical density (OD) as compared with the control 2, 3, and 4 weeks after the operation (2nd week: 0.249 ± 0.032 vs. 0.229 ± 0.039 vs. 0.191 ± 0.042; 3rd week: 0.246 ± 0.041 vs. 0.226 ± 0.043 vs. 0.212 ± 0.039; 4th week: 0.242 ± 0.038 vs. 0.225 ± 0.039 vs. 0.215 ± 0.042. *p* < 0.05 for all the analyses). Also, the BMP-2-positive expression was higher in the YNBY-treated group than that in the triiodomethane group and the control group 2 and 3 weeks after the operation (2nd week: 0.198 ± 0.061 vs. 0.113 ± 0.046 vs. 0.117 ± 0.051; 3rd week: 0.201 ± 0.041 vs. 0.161 ± 0.043 vs. 0.158 ± 0.039. *p* < 0.05 for all the analyses). Although this work preliminarily evaluates the healing effects of YNBY on the bone defects after cleft palate surgery, the mechanism of this formula is encouraged in the further study.

A recent study evaluated the healing effect of YNBY in human dental pulp cells (HDPCs) (Liu, 2014). The MTT result showed that YNBY at 10 μg/ml significantly promoted the cell proliferation as compared with the blank control. Thus, 10 μg/ml YNBY was used to induce the HDPCs. The von Kossa staining suggested that YNBY promoted obvious formation of mineralized nodules. Further, YNBY remarkably increased the OC protein expression on the 7th day and the Col-I and BSP mRNA levels on the 3rd, 7th, and 14th day after the induction.

In summary, this study confirms the healing effect of YNBY on the HDPCs induced by the mineralization-inducing media and preliminarily investigates the mechanism of this formula. However, the *in vitro* study is not enough to clarify the healing effects of YNBY and *in vivo* research is needed in the further study. Moreover, it is necessary to investigate changes in the pharmacodynamics of YNBY in the presence of specific blockers or siRNAs of pathways involving the target molecules such as Col-I and BSP.

Except for some growth-related factors as before (Liang, 2010; Liu, 2014), the roles of specific biophysical factors such as bioelectricity and ion fluxes in the wound healing have also been focused (Sun, 2015). Under anesthesia, two circular wounds (diameter 3 mm) were prepared on the back of a female C57BL/6 mouse using a 3-mm skin puncher. One hundred microliters of YNBY solution (1%, w/v) was added to a small piece of sterile absorbent cotton and then covered on the wound on one side. Correspondingly, the wound on the other side was covered with the sterile absorbent cotton immersed with an equal volume of sterile water. Compared with the control, YNBY significantly accelerated the wound healing 2, 6, and 10 days after the skin damage. Furthermore, the YNBY treatment markedly reduced the bioelectricity at the edge of the wound as compared with the control (−7.73 ± 0.73 vs. −23.38 ± 1.26 μA/cm^2^, *p* < 0.01). In addition, the ion fluxes including Na^+^, Ca^2+^, and Cl^−^ were significantly lower in the YNBY-treated group than those in the control group [Na^+^: -(1.44 ± 0.06) × 10^5^ vs. −(3.23 ± 0.25) × 10^5^ pmol/cm^−2^/s; Ca^2+^: −579.86 ± 104.73 vs. −1842.54 ± 187.89 pmol/cm^−2^/s; Cl^−^: (2.26 ± 0.28) × 10^5^ vs. (4.63 ± 0.55) × 10^5^ pmol/cm^−2^/s. *p* < 0.01 for all the analyses]. Taken together, this *in vivo* study confirms the healing effects of YNBY from a novel perspective. Although it is interesting, it still needs solid experiments to explain reasons for the changes in the currents and ion fluxes.

### Anti-Inflammatory Effects

The anti-inflammatory effects of YNBY contribute in eliminating swelling and alleviating pain, as well as eliminating necrotic tissues and promoting granulation. Suppressions of inflammatory cytokines and mediators such as TNF-α, IL-1β, IL-6, NO, and PGE2 are related to the anti-inflammatory effects (Lee et al., 2016; Wang G. et al., 2020; Wang et al., 2020).

A recent study evaluated the anti-inflammatory effects of YNBY in acute inflammation models in rats (Ren et al., 2017). In carrageenan-induced edema experiment, 96 SPF SD rats were randomly divided into a control group, a model group, a celecoxib group, and a YNBY group (*n* = 24). The animals in the celecoxib group and the YNBY group were respectively administrated with 10 mg/kg celecoxib or 50 mg/ml YNBY (1 ml) for seven consecutive days. Two hours after the last administrations, 0.15 ml carrageenan (150 µg) was subcutaneously injected into the left paw and 0.15 ml saline into the right paw as the control. The rats in the control group received no injections. The paw edema was measured at specific time points, and then the inhibition rate was calculated. The grouping of the AA-induced inflammation model (1 mg/ml AA, 0.1 mg/paw) was similar to the carrageenan-induced one except for the positive drug which was replaced by mizolastine (0.3 mg/kg). At the end of the experiment, the animals were sacrificed and the paws were collected for ELISA, histology, and qRT-PCR tests. The results demonstrated that YNBY significantly reduced carrageenan-induced paw edema by 12%, 285, 41%, and 29% at 1, 2, 3, and 4 h after the injections, respectively (*p* < 0.05 for all the time points). Similarly, YNBY markedly attenuated AA-induced paw edema by 9%, 13%, 14%, and 14% 1, 2, 3, and 4 h post the injections, respectively (*p* < 0.05 for all the time points). The qRT-PCR findings suggested that YNB remarkably downregulated the mRNA levels of cPLA2, FLAP, 5-LOX, and LTB4r as compared with the AA-induced model (*p* < 0.01 for all the analyses). In addition, the plasma protein levels of LTB4r, cPLA2, and 5-LOX were significantly lower in the YNBY-treated group than those in the AA-induced model group (*p* < 0.01 for all the analyses). Furthermore, YNBY markedly inhibited AA metabolite pathway key enzymes and inflammatory lipid mediators such as PGE2, cPLA2, and COX-2 at both mRNA and protein levels. Importantly, YNBY significantly inhibited AA- or carrageenan-induced inflammatory cell infiltration of paw tissue. It suggests that YNBY exerts its anti-inflammatory effects by modulating the COX and LOX pathways in AA metabolism. Actually, specific blockers of the COX and LOX pathways or gene knockout animals are needed to investigate the changes in anti-inflammatory effects of YNBY. In addition, it is inefficient to reflect the dose–efficacy relationship of YNBY for only a single dose of this formula was used in this study.

It revealed that YNBY reduced lipopolysaccharide (LPS)-induced inflammation in osteoclasts (Ren et al., 2020c). Briefly, the osteoclast cells were cultured with RPMI-1640 containing 10% FBS supplemented with 1% penicillin/streptomycin at 37°C in an atmosphere of 5% CO_2_. The three to five passage cells (10^5^/ml) were cultivated in six-well plates and incubated with FBS-free medium for 24 h. After being stimulated with LPS (1,000 pg/ml) for 12, 24, and 48 h, respectively, the cells were then treated with various concentrations of YNBY (10, 50, and 200 μg/ml). After that, pro-inflammatory cytokines, such as IL-1β, IL-6, and TNF-α, in the cell supernatant were determined by using mouse ELISA kits in accordance with the instructions of the manufacturer. The NO product was measured by the nitrate reduction method. In addition, IL-1β was used to stimulate the osteoclast cells to assay the effect of YNBY. Furthermore, NF-кB pathway inhibitor PDTC, MAPK pathway inhibitor SB20358, and the Wnt5a pathway-related miR101b were used to assess the roles of NF-kB, MAPK, and Wnt5a pathways in the anti-inflammatory effects of YNBY. The results showed that YNBY at 200 μg/ml markedly reduced the levels of IL-1β, IL-6, and TNF-α, while it had no significant suppression on the NO product. The qRT-PCR results revealed that YNBY remarkably downregulated the mRNA levels of COX-1, COX-2, A2, 5-LOX, and sEH and upregulated the CYP2C8 and CYP2C9 levels in dose- and time-dependent manners. This work systemically investigates the roles of MAPK/NF-кB/Wnt5a pathways in the anti-inflammatory activity of YNBY *in vitro*. Moreover, the *in vivo* validation experiments are needed in gene knockout animals in the future.

### Antioxidant Effects

Li et al. (2016a) evaluated the scavenging of DPPH free radicals and hydroxyl free radicals, as well as the reduction ability of YNBY. The result showed that the scavenging ability on DPPH free radicals was enhanced along with the increase in the concentration of YNBY. The scavenging rate of DPPH was 45.35% when YNBY arrived at 1 mg/ml. Also, YNBY had ability for scavenging hydroxyl free radicals. The scavenging rate of YNBY gradually increased from 0.8 mg/ml, and the rate reached 55.37% at 1 mg/ml. Also, YNBY at 1 mg/ml possessed a stronger scavenging ability than vitamin C. In addition, YNBY had certain reduction ability, but it was far weaker than vitamin C. YNBY effectively scavenges DPPH and hydroxyl free radicals, showing strong antioxidant activity. Further, it is necessary to extract active sites or isolate efficient ingredients with different solvents to compare the antioxidant activity systemically.

### Anti-Arthritis Effects

Although it has been recognized that rheumatoid arthritis (RA) is an autoimmune disease, it is closely related to inflammation (Ren et al., 2020a; Wei et al., 2020). The anti-arthritis effects of YNBY are linked to its activating blood circulation to dissipate blood stasis and eliminating swelling and alleviating pain.

Luo and Wang (2017) evaluated the anti-inflammatory effect of YNBY in the RA rat model induced by a model construction reagent (MCR) containing bovine type II collagen (CII) (2 mg/ml) dissolved in 0.1 mol/l acetate solution and the same volume of Freund’s complete adjuvant (FCA). A total of 128 SPF male SD rats weighing 220–250 g were randomly divided into four groups (*n* = 32). Ninety-six animals in the three groups were intracutaneously injected with 0.2 ml MCR on multiple sites in the root at the tails. In addition, 0.1 ml MCR was injected in the left rear voix pedis of each rat in the three groups. Next, 0.2 ml MCR was respectively injected again in the tail of each rat at days 7 and 21. The rats in the model group were given no treatment from days 7 to 35. The rats in the positive drug group received intragastric administrations of 1 ml MTX (0.2 mg/ml) once weekly for four consecutive weeks according to clinical practice. The animals in the YNBY-treated group were administrated with 1 ml YNBY (50 mg/ml) once daily for four consecutive weeks. The normal rats were given the same volume of physiological saline. The blood, urine, and swelled limbs were collected for measurements at the specific time points. The basic physical result indicated that YNBY significantly reduced the average voix pedis thickness of the affected limbs as compared with the model control (*p* < 0.01). Biochemical tests showed that YNBY markedly downregulated the serum IL-1β and PGE2 levels in the RA rats (*p* < 0.01; *p* < 0.01). YNBY significantly alleviated inflammatory cell infiltration, synovium hyperplasia, and narrowed joint space. YNBY at 20 μg/ml substantially increased intercellular but not extracellular PGE2 content in the osteoblasts. In summary, YNBY alleviates RA symptoms in rats, which is associated with the modulation of AA metabolism in the osteoblasts at some extent. However, some limitations are also present in this study. Firstly, it is more reasonable to allocate the animals into various groups after the successful establishment of the RA model. Secondly, the dose of YNBY used in the present study is inaccurate for it is impossible to use the same drug amount (50 mg) in individuals in the rats, as well as the use of MTX. Thirdly, it is difficult to reflect the dose–efficacy relationship of YNBY for only a single dose used. Moreover, the mechanism of YNBY against RA is not clearly clarified for the absence of gene knockout animal *in vivo* and specific blockers or siRNAs of related pathways *in vitro*.

### Antibacterial Effects

The antibacterial effects of YNBY support its eliminating necrotic tissues and promoting granulation.

Liu et al. (2019) evaluated the therapeutic effect of YNBY in *Staphylococcus aureus* (*S. aureus*)-induced hospital-acquired pressure ulcers (HAPU). The results revealed that the YNBY treatment for 20 days significantly reduced the wound area of the *S. aureus*-positive HAPU patients and improved the efficacy as compared with the control (*p* < 0.05; *p* < 0.05). The *in vitro* antibacterial results showed that the MIC and sub-MIC values of YNBY against ATCC29213 and *S. aureus* pALC1743, pALC1742, and pALC1740 were respectively 1 and 16 mg/ml, whereas the MICs of YNBY against *S. aureus* harboring genes coa, sarA, icaA, cidA, sea, seb, sec, sed, see, and tsst-1 were from 16 to 32 mg/ml, and the sub-MIC ranged from 1 to 2 mg/ml. The GFP-mediated fluorescence finding suggested that hla promoter activity was lower in the YNBY-treated pALC1740 than that in the untreated strain. Further, YNBY markedly lowered the overall fluorescence intensity and fluorescence intensity of the largest biofilm. Compared with the control, YNBY significantly inhibited coagulase activity and enterotoxin and tsst-1 as well as adhesion function-related genes’ expressions in *S. aureus*. In summary, YNBY reduces HAPU through inhibiting virulence genes’ expressions and the biofilm formation of *S. aureus*. However, related strains with gene deletion or overexpression are needed to clarify the mechanism of YNBY against HAPU.

Besides gram-positive bacteria, YNBY also suppressed gram-negative bacteria such as *Pseudomonas aeruginosa* (*P. aeruginosa*). Zhao (2013) evaluated inhibitory effects of the aqueous extract of YNBY on the quorum-sensing-related virulence of *P. aeruginosa*. The results indicated that the MIC and sub-MIC values of YNBY against *P. aeruginosa* were 55 and 2.5 mg/ml, respectively. Moreover, the extract at 3.0 mg/ml respectively reduced the cell density and total bacterial and extracellular protein by 13%, 13%, and 21%. Furthermore, YNBY at 2.5 mg/ml downregulated the transcriptional levels of *lasR*, *lasI*, *rhlR*, and *rhlI*, suggesting a quorum-sensing inhibitory (QSI) effect in a concentration-dependent manner. Similar to the QSI effect, YNBY at 2.5 mg/ml remarkably downregulated the mRNA levels of LasA protease, LasB elastase, and pyocyanin by 68.85%, 65.64%, and 76.47% in a concentration-dependent manner. Moreover, OdDHL, an autoinducer (AI) of the *las* system, significantly enhanced the inhibitory effect of YNBY on the three toxins. However, BHL, another AI of the *rhl* system, markedly antagonized the inhibitory effect of this formulae. YNBY (2.5 mg/ml) reduced the biofilm by 58.7%. Importantly, YNBY significantly reduced the motility of *P. aeruginosa*, which might be associated with the damaged flagella and TFP of this bacterium. Similar to the study of Liu et al. (2019), the validation experiments are necessary to clarify the mechanism of this formula against the gram-negative pathogenic bacterium in the presence of deletions or overexpressions of the target genes.

### Analgesic Effects

Pain is one of the most common symptoms accompanied with complex physiological and psychological activity. The activation of the NF-κB pathway and downstream cytokines such as TNF-α, IL-6, COX-2, and others has been confirmed in the initiation and development of pain (Singh, et al., 2020).

A study reported the analgesic effects of steroidal alkaloids from “Pi-ma-cao” (the roots and rhizomes of *Veratrum taliense* Loes.), the main analgesic components of YNBY (Li et al., 2016b). Multiple-step chromatography was used to isolate the total alkaloids. The structures of the compounds were elucidated by analyzing the spectroscopic data. Carrageenan-induced paw edema and acetic acid-induced writhing in mice were used to assessed anti-inflammatory and analgesic activities. It showed that three new alkaloids named veratraline A (**1′**), veratraline B (**2′**), and veratraline C (**3′**) together with eight known analogs including jervine (**4′**), 23-methoxy-cyclopamine (**5′**), dihydrojervine (**6′**), (1*β*,3*α*,5*β*)-1,3-dihydroxyjervanin-12-en-11-one (**7′**), and verdine (**8′**) were elucidated from the total alkaloids. Except for compound **8′**, all the other compounds significantly inhibited carrageenan-induced paw edema as compared with the control. Further, the compounds (**1′)** and (**3′)** at 0.5 mg/kg and the compounds (**2′)**, (**4′)**, (**5′)**, and (**7′)** at 1.0 mg/kg significantly inhibited the paw edema by 52.2, 67.1, 52.2, 58.2, 61.2, and 64.2%, respectively. All the compounds markedly decreased the number of writhes better than the positive control drug dolantin at 10 mg/kg. Unexpectedly, the writhing response was rapidly declined by 87.6%, 88.8%, 81.6%, and 91.7% after being treated by the compounds (**1′) and** (**3′)** at 0.5 mg/kg and (**5′)** and (**7′)** at 1.0 mg/kg, respectively. In summary, the compounds isolated from the total alkaloids in Pi-ma-cao exert strong anti-inflammatory and analgesic properties. The analgesic effects may be a potential research direction for this Chinese formula in the future. This study preliminarily investigates the analgesic effects of this plant, while it is encouraged to investigate the efficacy and analgesic mechanism of YNBY in pain animal models *in vivo*.

### Antitumor Effects

The antitumor effects of YNBY are linked to its activating blood circulation to dissipate blood stasis in the TCM concept.

Ji et al. (2001) evaluated the antitumor effects of YNBY, *Paris polyphylla* var. yunnanensis (Franch.) Hand.-Mazz. (PPM), gracillin, and methylmotogracillin *in vitro* by the MTT method. The result indicated that all the drugs tested exhibited antitumor effects against A-549, MCF-7, HT-29, A-496, PACA-2, and PC-3 cell lines, among which gracillin was best for all the 50% growth inhibition (GI_50_) values against these six tumor cell lines which were maintained at 10^−1^ μg/ml level. This study preliminarily assessed the *in vitro* antitumor effects of YNBY and its related plants and compounds.

Recently, a study evaluated the antitumor effects of YNBY in canine hemangiosarcoma (HSA) cell lines (Wirth et al., 2016). Various concentrations of YNBY (50, 100, 200, 400, 600, and 800 μg/ml) were used to treat three canine HSA cell lines including DEN-HSA, Fitz-HSA, and SB-HAS for 24, 48, and 72 h. After that, 50% inhibitory concentration (IC_50_), caspase-3/7 activity, and VEGF amount were determined. The IC_50_ values were respectively 369.9, 275.9, and 325.3 μg/ml for the DEN, Fitz, and SB cell lines at 72 h. The fluorescence density showed that YNBY (200, 400, 600 μg/ml) significantly increased the caspase-3/7 activity in the three cell lines in both concentration- and time-dependent manners. The FACS result demonstrated that YNBY ranging from 200 to 400 μg/ml produced the most significant change in the apoptosis percentage in these three cell lines. Cell cycle analysis showed that YNBY ranging from 50 to 400 μg/ml moderately increased G_1_ and G_2_ phases and decreased the S phase in the DEN cell line at 24 and 48 h. YNBY (50–400 μg/ml) moderately increased the G_2_ phase at 24, 48, and 72 h in both Fitz and SB cell lines. Compared with the baseline, YNBY significantly upregulated the VEGF levels (fold ×) at 72 h in the SB cell line (50 μg/ml: ×2.9 ± 1.1; 100 μg/ml: ×64.5 ± 3.7; 200 μg/ml: ×19.1 ± 1.9; 600 μg/ml: ×2.9 ± 0.4). In the SB cell line, YNBY at 100 μg/ml obtained its maximum increase in the VEGF level as compared with the control (3,170.0 ± 0.0 vs. 58.1 ± 2.2 pg/ml, *p* < 0.01). Similarly, the VEGF levels in the Fitz cell line were significantly higher in the YNBY-treated than in the control (100 μg/ml: ×3.7 ± 0.1; 200 μg/ml: ×4.5 ± 0.1; 600 μg/ml: ×3.8 ± 0.8; 800 μg/ml: ×4.0 ± 0.3). Overall, it suggests that YNBY promotes the HSA cell death in concentration- and time-dependent manners, which is associated with initiation of caspase-mediated apoptosis. Actually, the concentrations of YNBY used in the apoptosis experiments are equal to or higher than the IC_50_ values and unavoidably produced some cytotoxicity, which interferes with the accuracy of the results. Moreover, the therapeutic efficacy and mechanism *in vivo* of this formula are also needed.

### Sleep-Enhancing Effects

The sleep-enhancing effects of YNBY were evaluated by using a subthreshold hypnotic dose of pentobarbital sodium (Lu et al., 2014). Briefly, 40 ICR mice (half male and half female) weighing 18–20 g were randomly allocated into a control group and three YNBY-treated groups (0.3, 1.2, 4.8 g/kg) (*n* = 10). The animals in the groups were administrated only once. The mice in the control group were given equal volumes of 0.5% CMC-Na. Thirty minutes after the administrations, pentobarbital sodium (30 mg/kg) was intraperitoneally injected into each mouse. After that, the sleep onset latency and sleeping time (loss of righting reflex, LRR) of each animal were observed and recorded. The result showed that the three doses of YNBY had no significant effect on the sleep onset latency, while high-dose YNBY (4.8 g/kg) markedly prolonged the sleeping time as compared with the vehicle control. It suggests that YNBY enhances hypnotic effects of pentobarbital sodium at a subthreshold hypnotic dose, which provides a promising research direction for this Chinese formula in clinic in future.

### Safety Evaluation

Currently, the safety of YNBY has been evaluated in experimental animals and human beings.

General pharmacological studies on YNBY were carried out in mice and dogs (Lu et al., 2014). The result showed that YNBY significantly reduced the autonomous activities of mice in a dose-dependent manner. Compared with the vehicle control, YNBY at 4 and 8 g/kg synergistically prolonged the sleep time and markedly increased the number of electric shocks in mice. No significant effect on general behavior was observed in mice after being treated with YNBY. Additionally, YNBY had no obvious effects on the cardiovascular system, respiratory system, and body temperature of dogs. In conclusion, YNBY shows its safe use in dogs. Meanwhile, YNBY dose-dependently reduces the autonomous activities in mice. Moreover, it sensitizes the hypnotic effect of pentobarbital sodium and increases the tolerance to electric shocks in mice at some doses.

Recently, the embryo–fetus developmental toxicity of YNBY has been investigated in rats (Song et al., 2014). The results revealed that in pregnant rats high-dose YNBY (4 g/kg/d) for 10 consecutive days had certain maternal toxicity, including salivation, reduced activity, partly with sneezing or mouth scratching, and thick breath sounds. Increases in weights of YNBY-treated (0.5, 1.4, 4 g/kg/d) rats were slowed down. Food intake of the pregnant rats was lower in the middle- and high-dose YNBY groups (1.4, 4 g/kg/day) than in the control group, respectively. Compared with the control, the middle- and high-dose YNBY significantly reduced the body weights of fetal rats, while it had no obvious biological effects on embryo formation, fetal appearance, or visceral or skeletal development. Under this condition, YNBY had certain maternal toxicity in the pregnant rats but without significant embryo–fetal developmental toxicity and teratogenic effect. The developmental NOAEL of YNBY for rat embryos and fetuses was 0.5 g/kg. After that, Zhou et al. (2014) evaluated the toxicity of YNBY on fertility and early embryo development in rats. It suggested that from before mating to mating period to embryo implantation, the oral administration of YNBY had certain parental toxicity to both female and male rats but did not affect fertility and early embryonic development. The NOAEL for fertility and early embryo development toxicity was 4.0 g/kg in rats.

Currently, the clinical safety of YNBY has been evaluated in a large number of patients in various regions including Southwest, North, Northeast, and Central China (Xu et al., 2018; Yang et al., 2018; Song et al., 2019; Xiao et al., 2019). In these trials, the ADRs and adverse drug events (ADEs) of the included cases were assayed. No ADRs/ADEs were found when the YNBY capsules were used alone. The incidences of ADR/ADE of YNBY capsules were very low when combined with other drugs, and most were mild allergic reactions, gastrointestinal reactions, etc. They disappeared after drug withdrawal and dose reduction or taking other measures. Moreover, no new and serious ADRs/ADEs were found during the periods of these trials (Table 4).

**TABLE 4:** ADRs/ADEs of YNBY in a large number of patients.

Total number	ADRs/ADEs	Number of ADRs/ADEs	Incidence (%)	Severity	Outcomes	References
1,653	Cutaneous pruritus	1	0.06	Mild	Disappeared	Song et al. (2019)
	Gastrointestinal reactions	5	0.30	Mild to moderate	Disappeared	
	Chest tightness and palpitation	1	0.06	Moderate	Disappeared	
1,694	Gastrointestinal reactions	10	0.59	Mild	Disappeared	Xu et al. (2018)
	Cutaneous pruritus and local papula	3	0.18	Mild	Disappeared	
1,072	Diarrhea	1	0.09	Mild	Disappeared	Yang et al. (2018)
906	Stomach discomfort	2	0.22	Mild	Disappeared	Xiao et al. (2019)
	Papula	1	0.11	Mild	Disappeared	

## Clinical Applications

**FIGURE 4:**
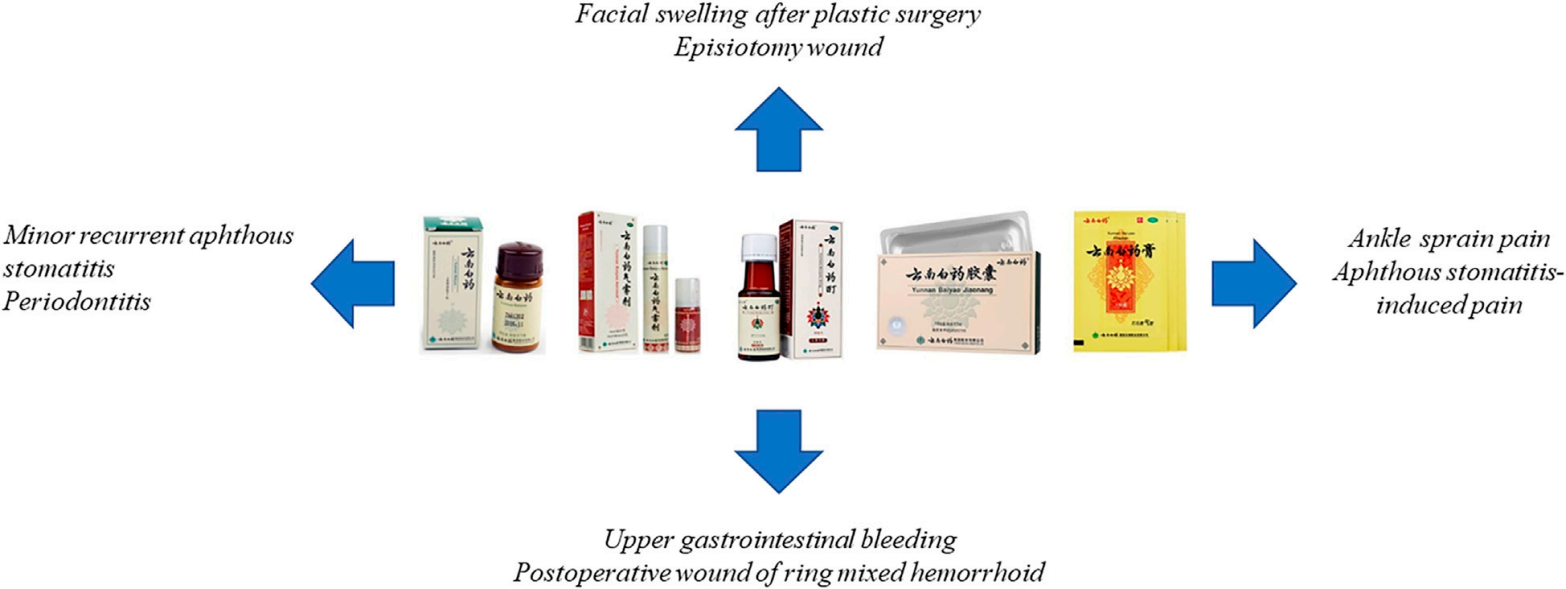
Clinical applications of Yunnan Baiyao preparations marketed in China.

Nowadays, the types of YNBY preparation in the Chinese market mainly include capsule, powder, aerosol, and paste (Figure 4).

Currently, YNBY has been widely used combined with other drugs or technologies in clinic to treat diseases mainly including bleeding (Zhao et al., 2018; Lin et al., 2019; Huang et al., 2020; Shi, 2020), swelling (Xiong et al., 2015; Wang and Yan, 2016), pain (Liu et al., 2012; Bo and Niu, 2012; Wu, 2016; Wang and Guan, 2019a), poor postoperative wound (Cao et al., 2017; Song et al., 2018), arthritis (Yuan and Qin, 2016; Luo and Wang, 2017), aphthous stomatitis (Liu et al., 2012), mouth ulcers (Tan, 2020; Ye et al., 2020), periodontitis (Wang, 2019b), ulcerative colitis (Zhao et al., 2016; Chen et al., 2019), skin ulcer (Yang et al., 2019b), and prostatitis (Huang et al., 2012; Cheng et al., 2015) (Table 5). However, it is rare for the clinical applications of YNBY alone. Thus, it is necessary to assess the efficacy of YNBY in treating diseases alone in the clinical trials.

**TABLE 5:** The clinical applications of YNBY.

Disease	Cases	YNBY dose (g)	Duration of YNBY	Combined drug/technology	Duration of combined drug	Response number	Response rate(%)	References
Upper gastrointestinal bleeding	50/50/50	0.25	Four times daily for 4 weeks	40 mg/kg omeprazole	Once daily for 4 weeks	48/37/27	96/74/54	Huang et al. (2020)
Upper gastrointestinal bleeding	26/26	0.25–0.50	Four times daily for 1 week	40 mg pantoprazole	Once daily for 1 week	24/16	92.31/61.54	Lin et al. (2019)
Liver cirrhosis complicated by upper gastrointestinal bleeding	75/75	1.0	Four times daily for 3 days	3.0 mg Somatostatin and 80 mg pantoprazole	Twice daily for 3 days	72/64	96.00/85.33	Zhao et al. (2018)
Facial swelling after plastic surgery	44/44	0.50	Four times daily for 1 week	Regular therapy and change of dressing	One daily for 2 weeks	42/34	95.54/77.27	Xiong et al. (2015)
Facial swelling after plastic surgery	25/25	0.50	Four times daily for 2 weeks	Regular therapy and change of dressing	One daily for 2 weeks	23/18	92/72	Wang and Guan, (2019a)
Ankle sprain pain	32/32	0.25–0.50	Once daily for 1 week	2.0–3.0 g lidocaine cream and elastic bandage for 3 weeks	Lidocaine for 1 week and bandage for 3 weeks	31/26	96.87/81.25	Bo and Niu, 2012
Aphthous stomatitis-induced pain	113/114	1 g toothpaste containing 0.0065 g active extract from YNBY	Twice daily for 5 days	—	—	28/17	66.4/51.8	Liu et al. (2012)
Postoperative wound of ring mixed hemorrhoid	58/58	An appropriate amount	Once to twice daily for 14 days	An appropriate amount dracon blood	Once to twice daily for 14 days	55/47	94.83/81.03	Song et al. (2018)
Episiotomy wound	100/100	An appropriate amount (Depth: 1–1.5 mm)	Three times daily for 5 days	0.5% iodophor	Three times daily for 5 days	99/91	99/91	Cao et al. (2017)
Osteoarthritis	50/50	0.50	Three times daily for 2 weeks	200 mg celecoxib	Twice daily for 2 weeks	47/40	94/80	Yuan and Qin, (2016)
Minor recurrent aphthous stomatitis	113/114	1 g toothpaste containing 0.0065 g active extract from YNBY	Twice daily for 5 days	—	—	31/18	27.4/15.8	Liu et al. (2012)
Mouth ulcers	30/30	An appropriate amount	Four times daily for 2 weeks	An appropriate amount of Shuangliao Houfeng power	Four times daily for 2 weeks	28/21	93.33/70	Ye et al. (2020)
Mouth ulcers	50/50	An appropriate amount	Five times daily for 1 month	5 mg vitamin B9, 5 mg vitamin B2, and 200 mg vitamin E	Twice daily for 1 month	49/41	98/82	Tan, (2020)
Periodontitis	50/50	0.25	Three times daily for 1 week	Regular therapy	Once daily for 1 week	48/41	94/82	Wang, (2019b)
Ulcerative colitis	30/30	0.50	Four times daily for 8 weeks	1–2 g sulfasalazine; 12–20 mg methylprednisolone	Sulfasalazine: 2–3 times daily for 8 weeks; methylprednisolone: once daily for 8 weeks	28/20	93.33/66.67	Chen et al. (2019)
Skin ulcer	25/24/24/23	An appropriate amount	Once daily for 4 weeks	Surgical dressing; water-filtered infrared-A	Surgical dressing: once daily for 4 weeks; water-filtered infrared-A 20 min once daily for weeks	23/17/16/10	92/70.83/66.67/43.48	Yang et al. (2019b)

## Conclusions—Current Research Status, Problems, Solutions, and Prospects

YNBY, as a TCM formula, has been used in China for over 100 years. Its main pharmacological effects include hemostasis, invigorating the circulation of blood, and wound healing.

Recent studies have suggested that above 0.5 g/kg this formula produces certain toxicology in rats. The clinical trials from various regions of China have confirmed the safety of YNBY in a large number of cases. Meanwhile, the ADRs/ADEs occur in very few patients. Currently, in clinic YNBY is widely used to treat bleeding caused by various factors such as gastric ulcer and perforation, operation, and trauma.

In the present study, the research advances in the pharmacology, safety, and clinical applications of YNBY were reviewed and analyzed. In addition, some issues were found during the review.

First, due to the national secret principle for the prescription of YNBY, the bioactive ingredients in this formula are not completely clarified yet. Thus, it is difficult to investigate the SARs of these active ingredients.

Second, the current pharmacology studies on YNBY mainly focus on the pharmacodynamics of this formula. Usually, the mechanisms in some studies as well as the following validation experiments are absent or incomplete. In addition, the quality controls including animal grouping, dose, duration, and positive drug are not strictly performed.

Third, the pharmacology studies on aqueous or alcohol extract of YNBY are few. If YNBY is a mixture of various TCMs, it is necessary to investigate the activity of the extracts by various polarity solvents. In addition, necessary chemical analysis including fingerprint by HPLC is needed.

Fourth, the preclinical safety evaluation is to be further carried out in primates such as monkey, although it has been done in mice, rats, and dogs.

Fifth, although the safety of YNBY has been performed in a large sample of patients, the efficacy of this formula against diseases still lacks in clinic.

According to the issues listed above, some solutions are raised to solve these questions above in the research of this formula.

(i) Extract active sites or isolate active ingredients from YNBY by using the chemical methods guided by pharmacological studies.
(ii) Analyze the SARs and enhance the bioactive effects by chemical modification based on the activities of the ingredients from the formulae.
(iii) Strictly abide and perform the quality controls in the pharmacological experiments. The mechanisms of YNBY are to be enhanced after the confirmation of the pharmacodynamics.
(iv) The safety of YNBY is also to be evaluated in more experimental animals as well as the pharmacokinetics of this formula *in vivo.*
(v) Evaluate the efficacy of YNBY in various diseases in clinic and expand its indications.
(vi) As a widely used Chinese formula, YNBY exerts its significant effect hemostasis. Except for this effect, the analgesic and sleep-enhancing effects may be promising and potential research directions for this formulae in the future.
(vii) Establish good agricultural practice (GAP) bases to control the quality of medicinal plants in the YNBY formulae.

Taken together, the present review provides a critical and comprehensive analysis on pharmacology, safety, and clinical applications of YNBY. In addition, some issues in the research and development of this formula are raised and solved in this paper. Further, the potential value of YNBY is highlighted and some new research directions are provided for this TCM formula for better exploration and exploitation in the future.

## Glossary

AA: arachidonic acid
ADE: adverse drug event
ADP: adenosine diphosphate
ADR: adverse drug reaction
ATCC: American Type Culture Collection
bFGF: basic fibroblast growth factor
BMBT: buccal mucosal bleeding times
BSP: bone sialoprotein
BSSC: blood stasis symptom-complex
CBC: complete blood count
CD: cluster of differentiation
CII: type II collagen
COX: Cyclooxygenase
cPLA2: cytosolic phospholipase A2
DMEM: Dulbecco’s modified eagle medium
DPPH: 2,2-diphenyl-1-picrylhydrazyl
FACS: fluorescence activated cell sorting
FCA: Freund’s complete adjuvant
FDA: food and drug administration
FLAP: arachidonate 5-lipoxygenase-activating protein
G: shear elastic modulus strength
**GI**_**50**_: 50% growth inhibition
GMP: α-granule membrane protein
GP: glycoprotein
HAPU: hospital-acquired pressure ulcers
HCT: hematocrit
HDPCs: human dental pulp cells
IC50: 50% inhibition concentration
ICR: institute of cancer research
IL: interleukin
5-LOX: 5-lipoxygenase
LPS: lipopolysaccharide
LTB4r: leukotriene B4 receptor
LY30: lysis at 30 min
MA: maximum amplitude
MAPK: mitogen-activated protein kinase
MCR: model construction reagent
MH: Mueller–Hinton
MIC: minimum inhibitory concentration
MTT: 3-(4,5)-dimethylthiahiazo (-z-y1)-3,5-di-phenytetrazoliumromide
MTX: methotrexate
NF-кB: nuclear factor-кB
NO: nitric oxide
NOAEL: non-toxic reaction dose
NMPA: National Medical Products Administration
OC: osteocalcin
OD: optical density
PGE2: prostaglandin E2
PMPTH: thrombin-stimulated release of PMP
PPM: *Paris polyphylla* var. yunnanensis (Franch.) Hand.-Mazz
PPP: platelet-poor plasma
PRP: platelet-rich plasma
PselTH: thrombin-stimulated P-selectin expression
PT: prothrombin time
qRT-PCR: quantitative polymerase chain reaction
RA: rheumatoid arthritis
RIA: radioimmunoassay
SAR: structure–activity relationships
SD: Sprague Dawley
TCM: traditional Chinese medicine
TEG: thromboelastography
TNF: tumor necrosis factor
tPACLT: tPA-induced clot lysis time
TT: thrombin time
VEGF: vascular endothelial growth factor
vWF: von Willebrand factor
YNBY: Yunnan Baiyao
